# Recombinant Production of Human Aquaporin-1 to an Exceptional High Membrane Density in *Saccharomyces cerevisiae*

**DOI:** 10.1371/journal.pone.0056431

**Published:** 2013-02-11

**Authors:** Julie Bomholt, Claus Hélix-Nielsen, Peter Scharff-Poulsen, Per Amstrup Pedersen

**Affiliations:** 1 Aquaporin A/S, Copenhagen, Denmark; 2 Department of Physics, Technical University of Denmark, Kongens Lyngby, Denmark; 3 Department of Biology, August Krogh Building, University of Copenhagen, Copenhagen, Denmark; Institute of Molecular and Cell Biology, Singapore

## Abstract

In the present paper we explored the capacity of yeast *Saccharomyces cerevisiae* as host for heterologous expression of human Aquaporin-1. Aquaporin-1 cDNA was expressed from a galactose inducible promoter situated on a plasmid with an adjustable copy number. Human Aquaporin-1 was C-terminally tagged with yeast enhanced GFP for quantification of functional expression, determination of sub-cellular localization, estimation of *in vivo* folding efficiency and establishment of a purification protocol. Aquaporin-1 was found to constitute 8.5 percent of total membrane protein content after expression at 15°C in a yeast host over-producing the Gal4p transcriptional activator and growth in amino acid supplemented minimal medium. In-gel fluorescence combined with western blotting showed that low accumulation of correctly folded recombinant Aquaporin-1 at 30°C was due to *in vivo* mal-folding. Reduction of the expression temperature to 15°C almost completely prevented Aquaporin-1 mal-folding. Bioimaging of live yeast cells revealed that recombinant Aquaporin-1 accumulated in the yeast plasma membrane. A detergent screen for solubilization revealed that CYMAL-5 was superior in solubilizing recombinant Aquaporin-1 and generated a monodisperse protein preparation. A single Ni-affinity chromatography step was used to obtain almost pure Aquaporin-1. Recombinant Aquaporin-1 produced in *S. cerevisiae* was not N-glycosylated in contrast to the protein found in human erythrocytes.

## Introduction

The nucleotide sequences of the rapidly increasing number of fully sequenced genomes have revealed that approximately one third of all genes in any kingdom encode integral membrane proteins [1]. The lipid nature of biological membranes prevents transport of most solutes between the cytosol and the extra cellular environment and between the cytosol and the interior of organelles. Cellular homeostasis therefore relies upon integral membrane proteins allowing selective trans-membrane movement of solutes and information. The essential physiological role of membrane protein activity is visualized by the fact that approximately 60% [2] of all approved drugs are targeting a membrane protein. Despite of this, by August 2012 the number of unique membrane protein structures has only reached 355 [http://blanco.biomol.uci.edu/Membrane_Proteins_xtal.html] which is extremely low compared to the more than 54,000 structures available through the Protein Data Bank [http:/www.rcsb.org/pdb/home/home.do]. Only 102 of these membrane proteins are from eukaryots including 36 of human origin. Heterologously expressed protein has been the starting point for seven of the structures [3], [4], [5], [6], [7], [8]. For essentially all membrane proteins their density in natural tissue is so low that purification on the milligram scale required for crystallization attempts is excluded. Access to purified membrane proteins is further complicated by the observation that the expression systems that successfully delivered more than 90% of proteins used for solving the structures for water soluble proteins, have failed in producing the required densities of recombinant membrane proteins. The reason for this is probably a general failure of cells to cope with high level expression of recombinant membrane embedded proteins [9], [10]. Very few examples are found in literature on heterologous expression of eukaryotic membrane proteins to a level where large-scale purification is straight forward or even possible [11], [12].

Aquaporins constitute a family of physiologically very important integral membrane proteins that are found in all three kingdoms, eubacteria, archaea and eukaryotes [13]. In human thirteen members of the aquaporin family have been identified [14]. As protein channels they all allow for passive transport of water [15] while some isoforms show additional permeability for small solutes like urea [16], glycerol [16], arsenite [17], antimonite [17], boric acid [18], silicic acid [19], nitrate [20], ammonia [21], hydrogen peroxide [22], carbon dioxide [23] or nitric oxide [24]. Human Aquaporin-1 (hAQP1) is a 269 amino acids long protein with six transmembrane segments. hAQP1 provides the plasma membranes of erythrocytes and proximal tubules of the kidney with a high water permeability allowing water to be transported along an osmotic gradient. The physiologically important function of AQP1 is underscored by the finding that AQP1 knock-out mice [25] and humans [26] with defective AQP1suffer from marked polyurea and show low urinary osmolality. AQP1 also plays an important role in choroid plexus epithelium [27] where it facilitates secretion of cerebrospinal fluid and intracranial pressure regulation [28]. AQP1 has been suggested to be involved in a number of pathophysiological conditions including migrane with aura [29], human renal disorders [30] and tumor angiogenesis [31] making it an interesting drug target.

All human Aquaporins have been heterologously expressed, primarily in *Xenopus* oocytes for characterization of their transport specificity. The highest reported production of hAQP1was obtained in *Pichia pastoris* [32] and reached 90 mg per liter growth medium. However, in this expression system the density (i.e. mg hAQP1/mg total membrane protein) of hAQP1 in crude membranes only amounted to 0.6% of total membrane protein content. hAQP1 has also previously been produced in *Saccharomyces cerevisia* at 0.5 mg per liter medium [33]. In insect cells the production levels of other aquaporin isoforms have reached between 0.5 and 3 mg per liter growth medium. The density of recombinant Aquaporin in crude membranes was not reported in these studies.

The challenge of the present paper has been to develop a *S. cerevisiae* based expression system and to identify conditions that substantially increase the membrane density of recombinant, functional hAQP1 beyond what has previously been reported and to develop an efficient and simple protein purification scheme. This was achieved by combining our previously described expression system [34] with the GFP tagging approach [12], [35], [36]. Access to large amounts of pure hAQP1 would expand the palette of screening tools aiming at identification of much requested specific inhibitors and modulators. Such compounds may show potential in diuretic-refractory edematous conditions like severe congestive heart failure, cancer and migraine with aura.

## Materials and Methods

### Yeast strains and culture conditions

Expression in S. cerevisiae was performed in strain PAP1500 ((α *ura3*-*52 trp1:: GAL10-GAL4 lys2*-*801 leu2*Δ*1 his3*Δ*200 pep4::HIS3 prb1*Δ*1.6R can1 GAL*) as described [34].

### Construction of hAQP1-GFP-8His expression plasmid

Human Aquoporin-1 was PCR amplified with AccuPol DNA polymerase (VWR, Denmark) and primers AQP1cerup (5' ACACAAATACACACACTAAATTACCGGATCAATTC-*TAAGATAATT* **ATGGCCAGCGAGTTCAAG** 3') and AQP1GFP (5' ACAACACCAGTGAATAATTCTTCACCTTTAGACAT**TTTGGGCTTCATCTCCACC**3') while yEGFP was PCR amplified using primers GFPup (5' **ATGTCTAAAGGTGAAGAATTAT** 3') and GFPHISdo (5' CTTCAATGCTATCATTTCCTTTGATATTGGATCATCTAATGGTGA-TGGTGATGGTGATGGTG**TTTGTACAATTCATCCATACCAT** 3'). Nucleotide sequences shown in bold are complementary to the template. The nucleotide sequence shown in italics in the hAQP1cerup primer is the Kozak sequence from the yeast *PMR1* gene. All other sequences are used for homologous recombination. The hAQP1-GFP-8His expression plasmid was generated by *in vivo* homologous recombination in *S. cerevisiae* by transforming the two PCR fragments along with *Bam*HI, *Hin*dIII digested pEMBLyex4 [37] into strain PAP1500.

### Membrane preparation

Yeast crude membranes were prepared by glass bead homogenization as described previously [38]. Briefly, yeast cells resuspended in lysis buffer (25 mM Imidazol, 1 mM EDTA, 1 mM EGTA, 10% (w/v) sucrose pH 7.5) containing 1 mM PMSF, 1 µg/ml Chymostatin, 1 µg/ml Pepstatin and 1 µg/ml Leupeptin were homogenized in a 50 ml tube for 4 times one minute. The homogenate was centrifuged at 3,000× g for 10 minutes at 4°C. The supernatant was centrifuged at 100,000× g for 1.5 hours to pellet the membranes.

### Protein quantification

The protein concentration in crude membranes was determined by the Lowry assay [39]. Briefly, 100 µl of crude membranes were incubated with 1 ml freshly made Lowry reagent (1.9% Na_2_CO_3_, 0.1 M NaOH, 0.01% CuSO_4_, 0.02% NaKC_4_H_4_O_6_• 4H_2_O) for 30 minutes before addition of 100 µl Folin - Ciocalteau reagent diluted 1∶1 in 18 mΩ water. OD_595_ was measured after additional 30 minutes incubation. 0 to 100 µg Ovalbumin was used to generate a linear standard curve.

### Deglycosylation

15 µg crude yeast membranes were incubated with 500 units Endo-H (New England Biolabs, USA) overnight at 4°C in lysis buffer (25 mM Imidazol, 1 mM EDTA, 1 mM EGTA, 10% (w/v) sucrose pH 7.5).

### Whole cell fluorescence

5 ml of yeast cells with a known optical density were harvested, washed in sterile water, re-suspended in 200 µl sterile water and transferred to a 96 well white microplate (Nunc, Denmark). Fluorescence was measured in a microplate reader (Fluoroskan Ascent, Thermo Scientific, USA) using water as a blank. Excitation was at 485 nm and emission at 520 nm.

#### Quantification of the membrane density of hAQP1-GFP proteins

A correlation was established between pmol GFP and fluorescence by mixing known molar amounts of purified histidine-tagged yeast enhanced GFP protein with 25 µg crude membranes from *S. cerevisiae* not expressing any GFP protein. This linear correlation was used to calculate the hAQP1-GFP content in 25 µg crude yeast membranes. Excitation in these experiments was at 485 nm and emission was at 520 nm.

Histidine-tagged yeast enhanced GFP was produced in *E. coli* BL21(DE3)pLysS from plasmid pET20bGFP-8His that was a generous gift from Dr. David Drew, Imperial College London, England. Histidine-tagged GFP was purified using Supplementary protocol 2 in [35].

### SDS-PAGE and western blotting

SDS-PAGE and western blotting were performed as previously described [34]. Briefly, membrane proteins were separated in 10% SDS-PAGE gels and transferred by semidry blotting to PDVF membranes. Western blots were developed using the Millipore Immobilon^TM^ Western chemiluminescent HRP substrate (Milipore, USA). Chemiluminescense was visualized using the Carestream Image Station 4000 MM (Kodak, USA). Anti-GFP-antibody was a generous gift from Dr. Jakob R. Winther, Department of Biology, University of Copenhagen. Polyclonal Horseradish Peroxidase conjugated pig anti-rabbit-antibody (P0217) was from DakoCytomation, Denmark.

### In-gel fluorescence

Membrane proteins were separated in 10% SDS-PAGE gels and in-gel fluorescence was measured using Carestream Image Station 4000 MM (Kodak, USA) under conditions described in [36]. Excitation was set at 465 nm and emission was measured at 535 nm.

For in-gel experiments fluorescence intensity of single protein bands was quantified using the ImageJ software [http://rsbweb.nih.gov/ij/]. Each protein band was treated like an individual particle, threshold was set to exclude the background and the integrated fluorescence intensity was determined by multiplication of area and fluorescence intensity of each protein band [40].

### Bioimaging of live yeast cells

*S. cerevisiae* cells used for bioimaging were grown and induced for Aquaporin-1-GFP production as described [38]. Briefly, having reached an OD_450_ = 1.0 cells were diluted in the same medium to OD_450_ = 0.05, grown to OD_450_ ≈ 0.5 and stained with FM4-64 [41] and DAPI [42]. Fluorescence was visualized at 1000× magnification with a Nikon Eclipse E600 fluorescence microscope equipped with an Optronics Magnafire model S99802 camera attached.

### Detergent solubilization of Aquaporin-1-GFP

Initial solubilization screens were performed using the popular detergent kit (D399-POP) from Affymetrix. This kit contains the six detergents CYMAL-5, Fos-Choline-12, n-Dodecyl-β-D- Maltoside, n-Decyl-β-D-Maltoside, CHAPS and n-Octyl-β-D-pyranoside. Crude membranes were incubated for 1 hour at 4°C with gentle reversal at a protein concentration of 2 mg/ml and a detergent: protein ratio of three in 50 mM phosphate buffer pH 7.5 containing 10 mM imidazole, 500 mM NaCl, 1 mM PMSF, 1 µg/ml Leupeptin, 1 µg/ml Pepstatin and 1 µg/ml Chymostatin. Solubilized and non-solubilized protein were separated by ultracentrifugation at 100,000× g at 4°C for 30 minutes. GFP fluorescence in the supernatant and the pellet was measured in white microplates (Nunc, Denmark) in a microplate reader (Fluoroskan Ascent, Thermo Scientific, USA) and used to quantify solubilization efficiency. Excitation in these experiments was at 485 nm and emission was at 520 nm.

### Ni-affinity purification of recombinant human Aquaporin1

CYMAL-5 solubilized Aquaporin-1 protein was diluted ten times in buffer A (50 mM phosphate and 500 mM NaCl pH 7.5) containing 10 mM imidazole and incubated overnight at 4°C with Ni-NTA Superflow (Qiagen, Germany). A CellThru Disposable Column (Clontech, USA) was packed with the Ni-NTA slurry. The column was washed with ten volumes of buffer A containing 10 mM imidazole and 0.75 mg/ml CYMAL-5, 30 volumes of buffer A with 30 mM imidazole and 0.75 mg/ml CYMAL-5, seven volumes of buffer A with 100 mM imidazole and 0.75 mg/ml CYMAL-5. Protein was eluted with three volumes of buffer A containing 250 mM imidazole and 0.75 mg/ml CYMAL-5 and subsequently with three volumes of buffer A with 500 mM imidazole and 0.75 mg/ml CYMAL-5. Eluted AQP1-GFP-8His protein was collected in fractions of 200 µl. All buffers contained 1 mM PMSF, 1 µg/ml Pepstatin, 1 µg/ml Chymostatin and 1 µg/ml Leupeptin.

### Fluorescence-detection size-exclusion chromatography (FSEC)

FSEC was carried out according to Kawate and Gouaux [43]. Briefly, membrane proteins at a concentration of 2 mg/ml were solubilized at a detergent: protein ratio of three in 50 mM phosphate buffer pH 7.5 containing 10 mM imidazole, 500 mM NaCl, 1 mM PMSF, 1 µg/ml Leupeptin, 1 µg/ml Pepstatin and 1 µg/ml Chymostatin for 1 hour at 4°C by gentle inversion of test tubes. Solubilized protein was isolated by centrifugation at 100,000× g in a Beckman Airfuge (Beckman, USA). Solubilized protein was separated on a Superose^TM^ 12 10/300 GL column using an Äkta purifier (GE Healthcare, USA). Fractions of 300 µl were collected from the column. GFP fluorescence in each fraction was measured in the Fluoroskan Ascent microplate reader (Thermo Scientific, USA) using an excitation wavelength of 485 nm and emission at 520 nm.

## Results

### Development of a *S. cerevisiae* expression system for human Aquaporin-1

**Figure 1:**
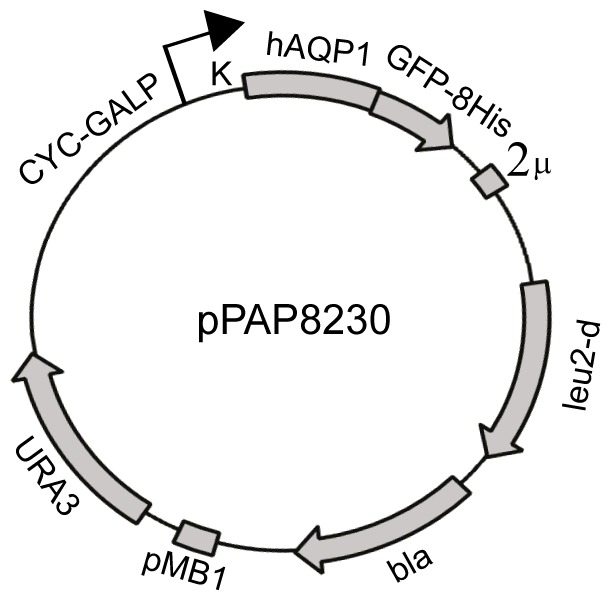
Structural map of the Aquaporin-1 expression plasmid pPAP8230. Abbreviations used: CYC-GALP, a hybrid promoter carrying the GAL10 upstream activating sequence fused to the 5' non-translated leader of the cytochrome-1 gene; K, Kozak sequence from the yeast *PMR1* gene; hAQP1, coding part of human aquaporin 1 cDNA without a translational stop codon; GFP-His, termination codon deficient yeast enhanced GFP cDNA fused in-frame to eight histidine codons; 2 µ, the yeast 2 micron origin of replication; *leu2-d*, a poorly expressed allele of the β-isopropylmalate dehydrogenase gene; *bla*, a ß-lactamase gene; pMB1, the pMB1 origin of replication; *URA3*, the orotinin-5'-P decarboxylase gene.

To investigate the capacity of *S. cerevisiae* for production of hAQP1 we constructed the expression plasmid pPAP8230 shown in Figure 1. Transcription of hAQP1-GFP-8His cDNA in PAP1500 *S. cerevisiae* cells transformed with pPAP8230 is driven by a CYC-GAL promoter and enhanced by application of a yeast strain overproducing the Gal4p transcriptional activator [34]. To potentially increase hAQP1 production the copy number of the expression plasmid can furthermore be increased ten times by selection for leucine autotrophy [44].

### Recombinant hAQP1-GFP accumulation depends on temperature and induction time

**Figure 2:**
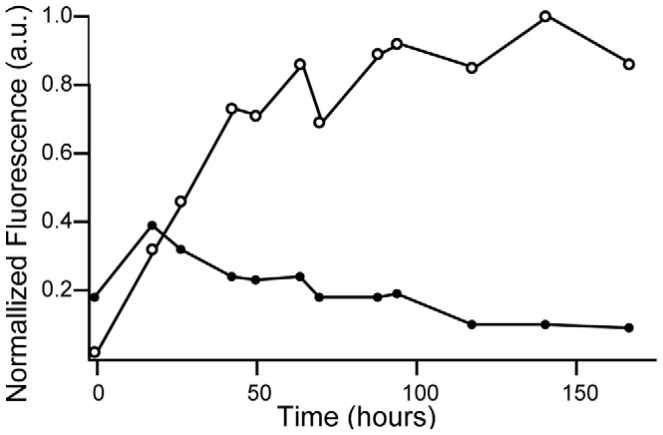
Time and temperature dependent accumulation of hAQP1-GFP in intact yeast cells. Briefly, yeast was inoculated in 2.5 liter shake flasks at room temperature to OD_450_ = 0.08 in 1 liter galactose-free expression medium (3% glycerol, 0.5% glucose minimal medium supplemented with all amino acids except leucine and isoleucine). At OD_450_ = 1.0 (time zero) half of the culture was transferred to 15°C and the other half to 30°C. Aquaporin expression was induced with 2% galactose 15 minutes later to assure that temperature equilibrium was obtained. Fluorescence was measured in intact yeast cells and normalized to cell number and the maximal fluorescence observed in the experiment. ○, induction of hAQP1-GFP production during growth at 15°C; •, induction of hAQP1-GFP production during growth at 30°C. Data is from a representative experiment.

The expression studies were performed in presence of all amino acids except leucine and isoleucine as we previously described these conditions to be beneficial for recombinant membrane protein accumulation [34]. Whole cell hAQP1-GFP fluorescence was used to determine the kinetics of accumulation of functional hAQP1with respect to induction temperature. Figure 2 shows that accumulation of hAQP1-GFP increased over time and reached a plateau after 60 hours of induction at 15°C, while accumulation at 30°C peaked shortly (≈12 hours) after induction and subsequently decreased. Expression at 15°C was therefore favorable for production of hAQP1-GFP.

### Reducing expression temperature to 15°C favors *in vivo* folding of hAQP1-GFP

**Figure 3:**
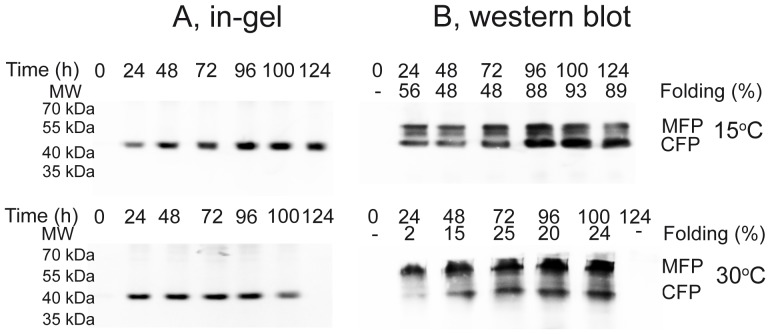
In-gel fluorescence and western bloting of yeast membranes. A, in-gel fluorescence of SDS-PAGE separated crude membranes isolated from yeast expressing the AQP1-GFP fusion for the times indicated at 15°C or 30°C. B, western blotting of the gels shown in ‘A’ using an anti-GFP-antibody. Folding efficiency at each time point and temperature was estimated as the ratio between the intensity of correctly folded hAQP-1-GFP fusion and the sum of intensities for the correctly folded and mal-folded GFP-fusions. MFP, mal-folded protein; CFP, correctly folded protein. The data are from a representative experiment.

To identify the molecular mechanism behind temperature sensitive accumulation of hAQP1-GFP we isolated membranes from yeast cells expressing the GFP fusion at either 15°C or 30°C and analyzed the purified membranes by in-gel fluorescence and western blotting. Only correctly folded GFP is visualized by in-gel fluorescence while correctly folded as well as mal-folded GFP are recognized by the anti-GFP-antibody in western blots. In the SDS-PAGE gel the Aquaporin-1 part of the fusion is denatured while the compact structure of correctly folded GFP is resistant to the applied SDS concentration [36]. The electrophoretic mobility of Aquaporin-1 fused to correctly folded GFP is therefore increased compared to that of Aquaporin-1 fused to mal-folded GFP. The in-gel fluorescence data in Figure 3A show that only a single membrane protein of approximately 40 kDa is visible after expression at 15°C and 30°C. The electrophoretic mobility of this band is in accordance with the expected molecular weight of the fluorescent band since hAQP1 has a molecular weight of 28.5 kDa and correctly folded GFP increases the molecular weight with 10–15 kDa [36] while the His-tag contributes with 1.1 kDa. The western blot data in Figure 3B show that the hAQP1-GFP-8His protein accumulated as a fast migrating correctly folded protein as well as a slower migrating mal-folded protein. Quantification of the data in Figure 3B show that up till 90% of hAQP-1 protein was correctly folded at 15°C while approximately 25% was correctly folded at 30°C.

### hAQP1-GFP accumulates to a very high density at 15°C

**Figure 4:**
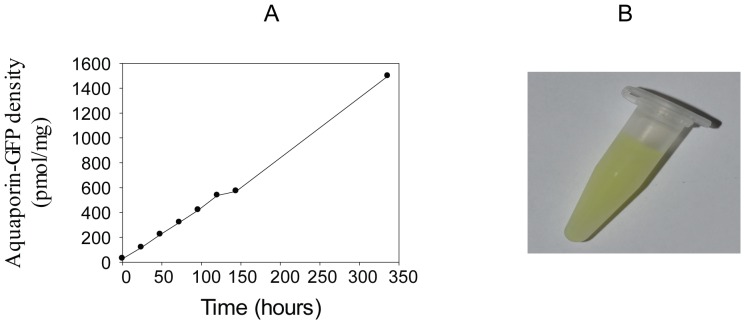
Time dependent accumulation of hAQP1-GFP fluorescence in crude membranes. **A**, hAQP1-GFP fluorescence in crude membranes isolated from yeast grown at 15°C at different time points after induction with galactose at time zero. Fluorescence intensity was converted to pmol GFP/mg crude membrane protein using a standard curve generated from purified yeGFP. **B**, crude membranes at a concentration of 6 mg/ml isolated 336 hours after induction with galactose.

Quantification of the in-gel fluorescence data in Figure 3A showed that correctly folded hAQP1-GFP accumulated in yeast membranes at 15°C even up till 124 hours after induction (data not shown). In order to determine if the membrane density of hAQP1-GFP protein kept increasing we quantified the time dependent accumulation of hAQP1-GFP produced at 15°C in crude membranes. It can be seen from Figure 4 that the density continued to increase for at least up till two weeks after induction, and that the density reached almost 1,500 pmol hAQP1-GFP per mg crude membranes. This corresponds to 8.5% of the total membrane protein content. The intense green color emitted from the crude membrane preparation shown in Figure 4 visualizes the high membrane density of the hAQP1-GFP fusion protein.

### Recombinant hAQP1-GFP is not N-glycosylated in *S. cerevisiae*

**Figure 5:**
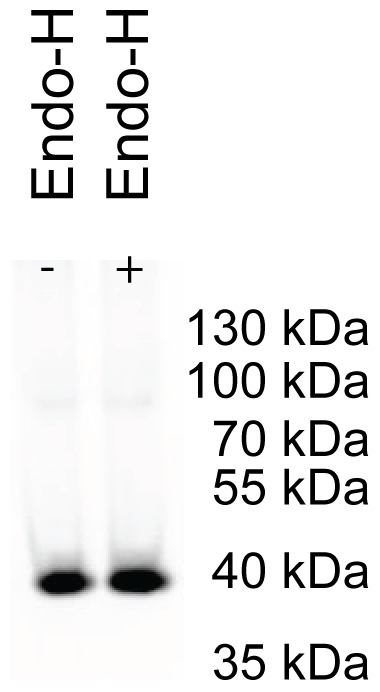
Endo glycosidase H treatment of yeast crude membranes. ÷, crude membranes from yeast producing hAQP1-GFP-8His; +, Endo-H treatment of crude membranes from yeast producing hAQP1-GFP-8His.

In erythrocytes hAQP1is found in two forms; a non-glycosylated version and an extensively N-glycosylated form [13]. To analyze whether recombinant hAQP1-GFP-8His is N-glycosylated we separated crude membranes treated or not with Endo-glycosidase H by SDS-PAGE an analyzed the outcome by in-gel fluorescence. The data in Figure 5 show that EndoH treatment did not affect the electrophoretic mobility of hAQP1-GFP-His8 showing that the fusion protein was not N-glycosylated.

### Recombinant hAQP1-GFP-8His is partly localized to the plasma membrane in yeast

**Figure 6:**
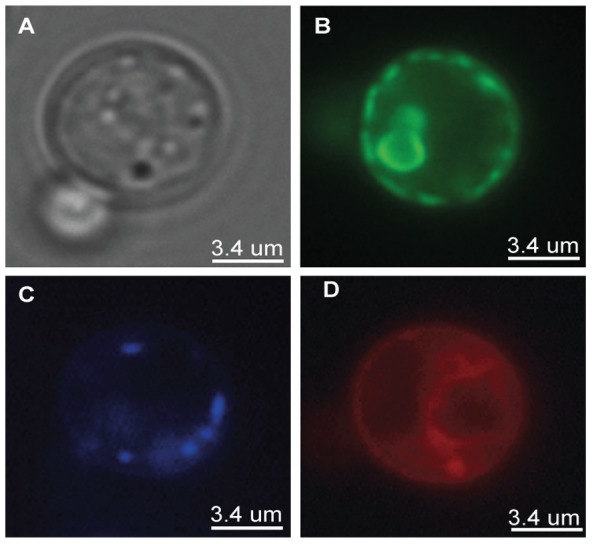
Live cell bioimaging of *S. cerevisiae* expressing GFP tagged hAQP1. (A), phase contrast; (B), GFP fluorescence; (C), DAPI fluorescence; (D), FM4-64 fluorescence. All images were generated at 100 times magnification.

Bioimaging of live yeast cells expressing hAQP1-GFP-8His was used to determine the sub cellular localization of the recombinant protein in yeast. Cells were additionally stained with DAPI to localize the nucleus and with FM4-64 that under the conditions used in the present protocol colors the vacuole as well as the plasma membrane. It can be seen from the micrographs in Figure 6 that a major part of hAQP1-GFP-8His was located non-uniformly in the plasma membrane; possibly indicating localization in lipid rafts. A part of the GFP fusion is also observed to localize in internal membranes, probably Endoplasmic Reticulum.

### Recombinant hAQP1-GFP-8His can be solubilized in detergent

**Figure 7:**
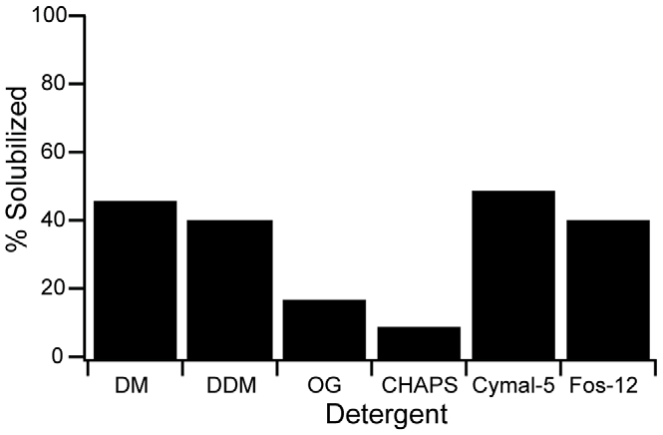
Detergent screen for solubilization of hAQP1-GFP-8His. Crude membranes were solubilized as described in Materials and Methods. GFP fluorescence was used to calculate percent solubilized hAQP1-GFP-8His. Abbreviations used; DM, n-decyl-β-D-maltopyranoside; DDM, n-dodecyl-β -D-maltopyranoside; OG, n-octyl-β -D-glucopyranoside; CHAPS, 3-[(3-Cholamidopropyl)-Dimethylammonio]-1-Propane Sulfonate/N,N-Dimethyl-3-Sulfo-N-[3-[[3α,5β,7α,12α)-3,7,12-Trihydroxy-24-Oxocholan-24-yl]Amino]propyl]-1-Propanaminium Hydroxide; CYMAL-5, 5-Cyclohexyl-1-Pentyl-β-D-Maltoside; Fos-12, n-Dodecylphosphocholine.

In order to purify the hAQP1-GFP-8His fusion protein we performed a detergent screen using six detergents commonly used for membrane protein purification. Data in Figure 7 show that CYMAL-5 was the most efficient detergent for solubilization of recombinant hAQP1-GFP-8His closely followed by DDM.

### Solubilized recombinant hAQP1-GFP-8His is monodisperse and mainly exists as a tetramer

**Figure 8:**
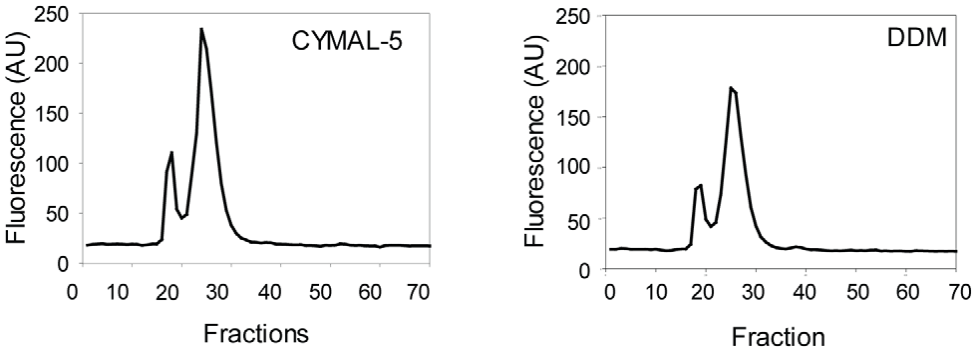
Fluorescent-detection size-exclusion chromatography of hAQP1-GFP-8His solubilized in CYMAL-5 or DDM. Crude membranes solubilized in CYMAL-5 or DDM at a detergent: protein ratio of 3 were separated by HPLC on a Superose^TM^ 12 10/300 GL column. Fractions of 300 µl were collected from the column and analyzed for GFP fluorescence as described in Materials and Methods.

A suitable detergent efficiently solubilizes the membrane protein of interest, gives a monodisperse protein preparation and maintains protein stability over a long period of time. To analyze for these quality criteria we examined the fusion protein by fluorescent size exclusion chromatography (FSEC) [43] of hAQP1-GFP-8His solubilized in CYMAL-5 or DDM to analyze for monodispersity. Data in Figure 8 show that the two detergents gave rise to similar chromatograms; a prominent symmetrical peak eluting in a total volume of less than 2 ml indicating a monodisperse protein preparation [35]. The elution volume corresponds to a molecular weight of approximately 200 kDa and indicates that solubilized, recombinant hAQP1-GFP-8His mainly exists as a tetramer in both detergents. Presence of a minor symmetrical peak shows that hAQP1-GFP-8His oligomers with a stoichiometry larger than four also exist. There is no sign of protein degradation as no free-GFP peak is visible.

### CYMAL-5 solubilized Aquaporin1 can be purified by Ni^2+^-affinity purification

**Figure 9:**
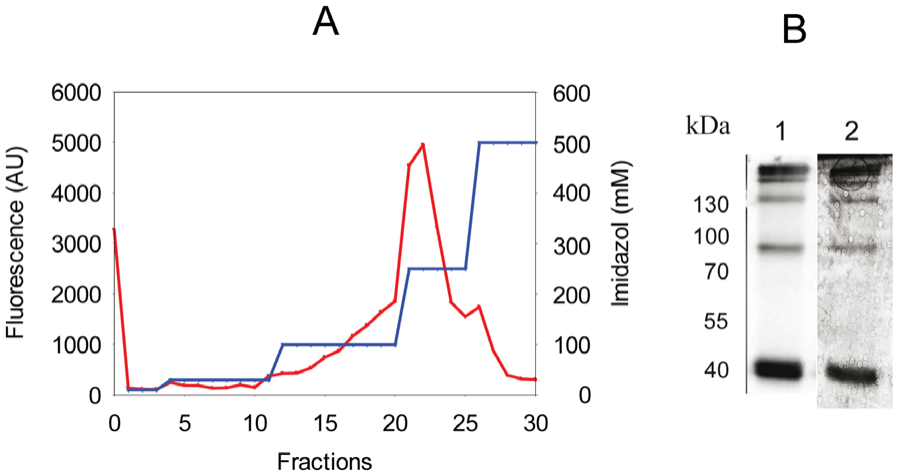
Affinity purification of hAQP1-GFP-8His. Crude membranes were solubilized in CYMAL-5 and purified by Ni-affinity chromatography as described in Materials and Methods. **A**, GFP fluorescence (red) was used to quantify the amount of hAQP1 in each fraction. The Imidazol profile used to wash and elute protein from the Ni-column is shown in blue. AU, arbitrary fluorescence units. **B**, (1) in-gel fluorescence after SDS-PAGE separation of the protein content of fraction 22; (2), Coomassie staining of the SDS-PAGE gel used for in-gel fluorescence in panel (1). Fraction 0, flow-through; fractions 1- 3, wash with 10 mM Imidazole; fractions 4–11 wash with 30 mM Imidazole; fractions 12–20, wash with 100 mM Imidazole; fractions 21–25, wash with 250 mM Imidazole; fractions 26–30, wash with 500 mM Imidazole.

CYMAL-5 efficiently solubilized hAQP1-GFP-His8 and produced a monodisperse protein preparation mainly consisting of the native tetrameric structure. We therefore selected CYMAL-5 solubilized hAQP1-GFP-8His for purification by affinity chromatography. A chromatogram resulting from the purification procedure is shown in Figure 9A. Data from the purification protocol revealed that 86% of the CYMAL-5 solubilized hAQP1-GFP-8His protein bound to the Ni^2+^-resin and 62% of the solubilized and bound material was eluted with 250 mM imidazole. The peak-fraction collected after elution with 250 mM imidazole was analyzed by SDS-PAGE separation using in-gel fluorescence and Coomassie staining, Figure 9B. As expected monomeric, dimeric, trimeric as well as tetrameric hAQP1-GFP-8His proteins were visible. The Coomassie staining furthermore showed that solubilization of the hAQP1-GFP-8His protein by CYMAL-5 followed by Ni-affinity chromatography resulted in highly pure human Aquaporin-1 protein. Very importantly only protein bands visualized by in-gel fluorescence were observed in the Coomassie stain. None of the slower migrating, non-fluorescent and mal-folded hAQP1-GFP-8His proteins observed in the western blot in Figure 3 were present in the purified sample.

## Discussion

The present paper describes an expression system and expression conditions that produce a very high membrane density of heterologously expressed human Aquaporin 1. It is approximately fourteen times higher than previously described [32] and to our knowledge the membrane density obtained in this study is unprecedented for a recombinantly produced human membrane protein. The potential of the present expression system for structure-function and structural studies of mammalian membrane proteins is obvious when compared to the densities of recombinant membrane protein previously serving as starting point for purification and crystallization of mammalian membrane proteins. The densities obtained for 7TM receptors were all in the range of 50 pmol/mg total membrane protein (corresponding to 0.2% of total membrane protein content) after expression in *P. pastoris* or insect cells [6], [45]. The expression system described here is able to deliver around 1,500 pmol hAQP1-GFP/mg total membrane protein corresponding to 8.5% of total membrane protein content. A high membrane density in the starting material is a significant advantage for purification of large amounts of recombinant membrane protein. Using our previously described fermentation setup [34] the present expression system may deliver in the range of 350 mg human Aquaporin-1 per 200 grams of yeast cells after a single fermentation.

To ease quantification of correctly folded hAQP1, sub-cellular localization, *in vivo* folding efficiency and development of a purification protocol, we chose to produce hAQP1 C-terminally tagged with GFP and an eight histidines long purification tag, a concept previously described [46]. The expression system described in the present paper extends work initially developed for recombinant production of the α_1_,β_1_ pig kidney Na,K-ATPase [34]. In agreement with these results the combined use of a galactose inducible promoter, an expression plasmid with an adjustable copy number, a protease deficient yeast host strain selectively overproducing the Gal4p transcriptional activator during the protein production phase and growth in amino acid supplemented minimal medium turned out to be beneficial for membrane accumulation of correctly folded hAQP1-GFP. Reducing growth temperature from 30°C to 15°C during the protein production phase has sometimes turned out to be advantageous for accumulation of recombinant membrane proteins [47] probably due to increased folding efficiency at the lower temperature. Production of hAQP1-GFP at 15°C was also superior for accumulation of correctly folded hAQP1-GFP fusion as can be seen from Figure 2. Whole cell fluorescence showed that accumulation at 30°C peaked around 24 hours after galactose induction and subsequently declined. In contrast when measured as whole cell fluorescence accumulation of hAQP1-GFP at 15°C was stabilized at a plateau after approximately fifty hours. The C-terminal GFP allowed us to determine the molecular mechanism behind the observed temperature sensitive accumulation. As described [36] C-terminal GFP only folds into a biologically active conformation if the fusion partner folds correctly. A fast migrating hAQP1-GFP fusion protein carrying a compact and correctly folded GFP can therefore be separated from a slower migrating hAQP1-GFP fusion protein carrying denatured GFP by SDS-PAGE. Only a hAQP1-GFP fusion carrying correctly folded GFP is visualized by in-gel fluorescence, while western blotting using an anti-GFP-antibody visualizes hAQP1-GFP fusion protein independent of GFP folding. Visual inspection of our data in Figure 4 clearly shows that folding of hAQP1-GFP is far more favorable at 15°C than at 30°C as the hAQP1-GFP band carrying correctly folded GFP dominated at 15°C while the hAQP1-GFP band representing mal-folded GFP dominated at 30°C. Quantification of the western blot bands confirmed that approximately 90% of the accumulated hAQP1-GFP protein was correctly folded at 15°C whereas only approximately 20% was correctly folded at 30°C.

We used GFP fluorescence to optimize expression and to quantify the density of hAQP1-GFP in yeast membranes. During this process we discovered a discrepancy between fluorescence from intact yeast cells producing hAQP1-GFP and fluorescence from purified crude membranes. Fluorescence from intact yeast cells reached a stable plateau after approximately fifty hours of induction at 15°C while fluorescence from purified crude membranes showed a linear increase in fluorescence with time even after two weeks induction with galactose. This discrepancy may be a result of the fluorescence is quenched by the intact cells. Quenching was also observed when analyzing fluorescence from crude membranes (data not shown), indicating that a rather low concentration of membranes must be used for quantification of fluorescence. Thus purified GFP used for generating a standard curve should be mixed with the same concentration of non-fluorescent membranes, to avoid underestimation of the membrane density of the GFP tagged protein.

In human tissue hAQP1 is localized to the plasma membrane. This localization seemed to be preserved in *S. cerevisiae* as the hAQP1-GFP fusion protein accumulated primarily in the plasma membrane in patches that may represent lipid rafts. Localization of the fusion protein to the plasma membrane is also a strong indication of a correct three dimensional structure as mal-folding would prevent it from leaving the ER and would prohibit it from becoming fluorescent.

Finding a detergent that efficiently solubilizes the hAQP1-GFP-8His fusion is essential for establishing an efficient purification protocol. Previous purification protocols for AQP1 from either *S. cerevisiae* [33] or *P. pastoris* [32] used β-OG for solubilization while purification from erythrocytes involved Triton-X 100 [13]. Our detergent screen revealed that CYMAL-5 was the most efficient yielding 50% solubilization of the fusion protein. The solubilization efficiency was slightly lower for DM, DDM and Fos-cholin 12, while OG and CHAPS were the most inefficient. We therefore selected CYMAL-5, which to our knowledge has not previously been used for solubilization and purification of hAQP1.

Presence of the GFP tag allowed us to monitor and quantify purification efficiency. From the data in Figure 7 it can be seen that 62% of the solubilized hAQP1-GFP that bound to the Ni-column was eluted at an imidazol concentration of 250 mM. In-gel fluorescence followed by Coomassie staining showed that the protein eluted as a monomer, dimer, trimer and tetramer as seen for purification of the native protein from erythrocytes [48]. The Coomassie stain shows that solubilization in CYMAL-5 followed by Ni-affinity chromatography resulted in a very pure preparation of recombinant hAQP1-GFP-8His fusion protein. Comparing the in-gel fluorescence with the Coomassie stain (Figure 7) also indicates that the purified hAQP1-GFP-8His fusion proteins are correctly folded since only bands detected by in-gel fluorescence were visible in the Coomassie stain. The slower migrating and non-fluorescent hAQP1-GFP-8His fusion proteins present in the western blot in Figure 3 were absent in the purified preparation. In contrast to Aquaporin-1 from erythrocytes we showed that the recombinantly produced protein in yeast was not N-glycosylated.

In conclusion we have developed an expression system that substantially increases the membrane density of recombinant hAQP1.This expression system enables low cost production of large amounts of functional protein for structural and biophysical studies and may become an important tool for identification of hAQP1 modulators.
